# Diffusion kurtosis imaging and diffusion weighted imaging comparison in diagnosis of early hypoxic–ischemic brain edema

**DOI:** 10.1186/s40001-023-01090-x

**Published:** 2023-05-03

**Authors:** Yuxuan Han, Peng Wu, Juan Tian, Honghai Chen, Chao Yang

**Affiliations:** Department of Radiology, The Second Affiliated Hospital of Dalian Medical University, No. 467, Zhongshan Road, Shahekou District, Dalian, Liaoning Province China

**Keywords:** Hypoxic–ischaemic encephalopathy, Apparent diffusion coefficient, Diffusion kurtosis imaging, Mean diffusion coefficient, Piglets

## Abstract

**Background:**

Hypoxic–ischemic encephalopathy (HIE) refers to cerebral hypoxic–ischemic injury caused by asphyxia during perinatal period, which is one of the important causes of neonatal death and sequelae. Early and accurate diagnosis of HIE is of great significance for the prognostic evaluation of patients. The purpose of this study is to explore the efficacy of diffusion-kurtosis imaging (DKI) and diffusion-weighted imaging (DWI) in the diagnosis of early HIE.

**Methods:**

Twenty Yorkshire newborn piglets (3–5 days) were randomly divided into control group and experimental group. DWI and DKI scanning were performed at timepoints of 3, 6, 9, 12, 16, and 24 h after hypoxic–ischemic exposure. At each timepoint, the parameter values obtained by each group scan were measured, and the lesion area of the apparent diffusion coefficient (ADC) map and mean diffusion coefficient (MDC) map were measured. (For better interpretation of this study, we replaced the description of MD with MDC). Then, we completely removed the brain for pathological examination, and observed the state of cells and mitochondria in the ADC/MDC matching area (the actual area of the lesion), and the mismatch area (the area around the lesion).

**Results:**

In the experimental group, the ADC and MDC values decreased with time, but the MDC decreased more significantly and the change rate was higher. Both MDC and ADC values changed rapidly from 3 to 12 h and slowly from 12 to 24 h. The MDC and ADC images showed obvious lesions at 3 h for the first time. At this time, the area of ADC lesions was larger than that of MDC. As the lesions developed, the area of ADC maps was always larger than that of the MDC maps within 24 h. By observing the microstructure of the tissues by light microscopy, we found that the ADC and MDC matching area in the experimental group showed swelling of neurons, infiltration of inflammatory cells, and local necrotic lesions. Consistent with the observation under light microscope, pathological changes were observed in the matching ADC and MDC regions under electron microscopy as well, including collapse of mitochondrial membrane, fracture of partial mitochondrial ridge, and emergence of autophagosomes. In the mismatching region, the above pathological changes were not observed in the corresponding region of the ADC map.

**Conclusions:**

DKI’s characteristic parameter MDC is better than ADC (parameter of DWI) to reflect the real area of the lesion. Therefore, DKI is superior to DWI in diagnosing early HIE.

## Introduction

Hypoxic–ischaemic encephalopathy (HIE) refers to cerebral hypoxic–ischaemic damage caused by asphyxia during the perinatal period, which is one of the most important reasons of neonatal death and sequelae. Therefore, an early accurate diagnosis of HIE has important practical significance for the choice of treatment plan and evaluation of prognosis [1–4]. The diffusion-weighted imaging (DWI) is widely used in the imaging method for diagnosing HIE. It is based on the premise that the diffusion of water molecules in brain tissue follows Gaussian distribution [5–7]. Its characteristic parameter apparent diffusion coefficient (ADC) can reflect the diffusion rate of water molecules, and indirectly reflect the changes in the microstructure and movement of water molecules inside and outside cells and extracellular matrix. In fact, the complex internal and external environment of the human body under hypoxic condition deviates considerably from the physiological diffusion of water molecules which were originally normally distributed in normoxyemic condition. The diffusion-kurtosis imaging (DKI) is a new magnetic resonance diffusion imaging technology, which can more truly reflect the diffusion motion of water molecules with non-Gaussian distribution [8]. It has previously been shown that the complexity of the microstructure of reactive tissues is much more reliably represented on DKI than on DWI [9]. The two most typical parameters of DKI are mean kurtosis (MK) and mean diffusion coefficient (MDC). MK is an indicator that reflects the complexity of tissue microstructure [10–12], which is widely considered to be the most sensitive parameter for DKI to evaluate ischemic lesions. MDC is a comprehensive indicator which asses the diffusion status of a voxel, which reflects the overall situation of molecular diffusion level and diffusion resistance in the ischemic tissues, and can be regarded as ADC corrected by non-Gaussian distribution [13, 14]. In addition, piglets and humans have a high degree of similarity in terms of gene sequence, chromosomes and anatomical structure. Therefore, newborn piglets can be used as a model to better mimick human HIE. Our study attempted to compare the value of DWI and DKI in MRI imaging for the early diagnosis of HIE.

## Materials and methods

### Animal experiments

Twenty healthy Yorkshire piglets (11 male, 9 female, 3–5 days, weighing 1.5 ± 0.2 kg) were randomly divided into control group (*n* = 5; 3 male, 2 female) and experimental group (*n* = 15; 8 male, 7 female), which came from the Dalian Hua-qiao Chinese Pig Farm. The experimental group was anesthetized with isoflurane, ligated bilaterally the common carotid arteries, and then suture the incision. After resuscitation, the piglet was placed in a tank, and a mixed gas (2 L/min) consisting of 4% oxygen and 96% nitrogen was delivered for 30 min. In the control group, only skin incision and blood vessel separation were performed, and bilateral common carotid artery ligation and hypoxia were not performed. Gentamicin was given 3 h before and after the operation to prevent infection.

All animal experiments were approved by the local institutional animal protection and use committee and complied with the National Institutes of Health guidelines.

### Equipment and methods

Using GE 3.0-T Discovery MR750w scanner and 32-channel magnetic head coil, using coronal scanning, T1WI, T2WI, DWI and DKI were obtained. The DWI scanning parameters were repetition time (TR)/echo time (TE) = 4500 ms/min, field of view (FOV) = 220 × 220 mm, matrix = 128 × 128, number of layers/layer thickness/layer spacing = 18/3.0 mm/0.5 mm, two b values in each direction (0, 1000 s/mm^2^), and scanning time = 95 s. The DKI scanning parameters were TR/TE = 4500 ms/min, FOV = 220 × 220 mm, matrix = 128 × 128, NEX = 2, and number of layers/layer thickness/layer spacing = 18/3.0 mm/0.5 mm. The direction of the diffusion-sensitive gradient field was 20, there were three b values in each direction (0, 1000, 2000s/mm^2^), and the scanning time was 350 s. An anchor made of cardboard holds the piglet in a prone position. Under anesthesia, the experimental and control groups underwent MR scanning at 3, 6, 9, 12, 16, and 24 h.

### Image post-processing

We used FuncTool on the GE AW4.7 workstation to post-process all raw data. Parameter images related to DKI and DWI can be obtained at the same time. Parameter maps of MK, MDC, ADC, axial kurtosis (AK), and radial kurtosis (RK) were obtained. In the experimental group, the region of interest (ROI, area = 2 mm^2^) was placed, where MK, ADC, and MDC values changed most significantly and in the corresponding area of the control group. Image J software (https://imagej.nih.gov/ij/) was used to measure the lesion area at the same level on ADC and MDC images at various timepoints, and the average value was obtained after three measurements. The above operations were performed by two experienced radiologists. A third radiologist examined the results.

### Histopathological diagnosis

After the 24 h scan, the inhaled anesthesia experimental group and control group were fixed on the operating table in a prone position. The brain tissue was completely removed by craniotomy. According to the ADC and MDC images, the lesion layer was measured with a ruler starting from the edge of the brain tissue, and the ADC and MDC matching and mismatching areas were measured, respectively. The layer corresponding to the match or mismatch areas were cut with a sterile blade into a layer with a thickness of about 3 mm and fixed in a 4% paraformaldehyde solution. After conventional dehydration, xylene transparency, and paraffin embedding, the sections were observed by light microscope. ADC/MDC matching and mismatching regions were taken from brain tissue with a volume of about 1 mm^3^ in glutaraldehyde solution, and fixed sections were stained with uranyl acetate and citric acid, then passed on a transmission electron microscopy (TEM) for observation. Two experienced pathologists evaluated histological changes and took pictures of typical lesions.

### Statistical analysis

All statistical analyses were performed using SPSS 20.0 software (SPSS, Chicago, IL, USA). The normality and variance uniformity of the obtained parameter values were tested, and the data were expressed as mean ± standard deviation. Repeated measures analysis of variance were used to calculate the difference in parameter values between different timepoints and between groups, and the *t* test was used to compare the percentage changes of different indicators within and between groups. All comparisons were made using the least significant difference method after the fact. *P* < 0.05 was considered statistically significant.

## Results

### Signal changes of DWI and DKI-derived variables in lesions

**Fig. 1:**
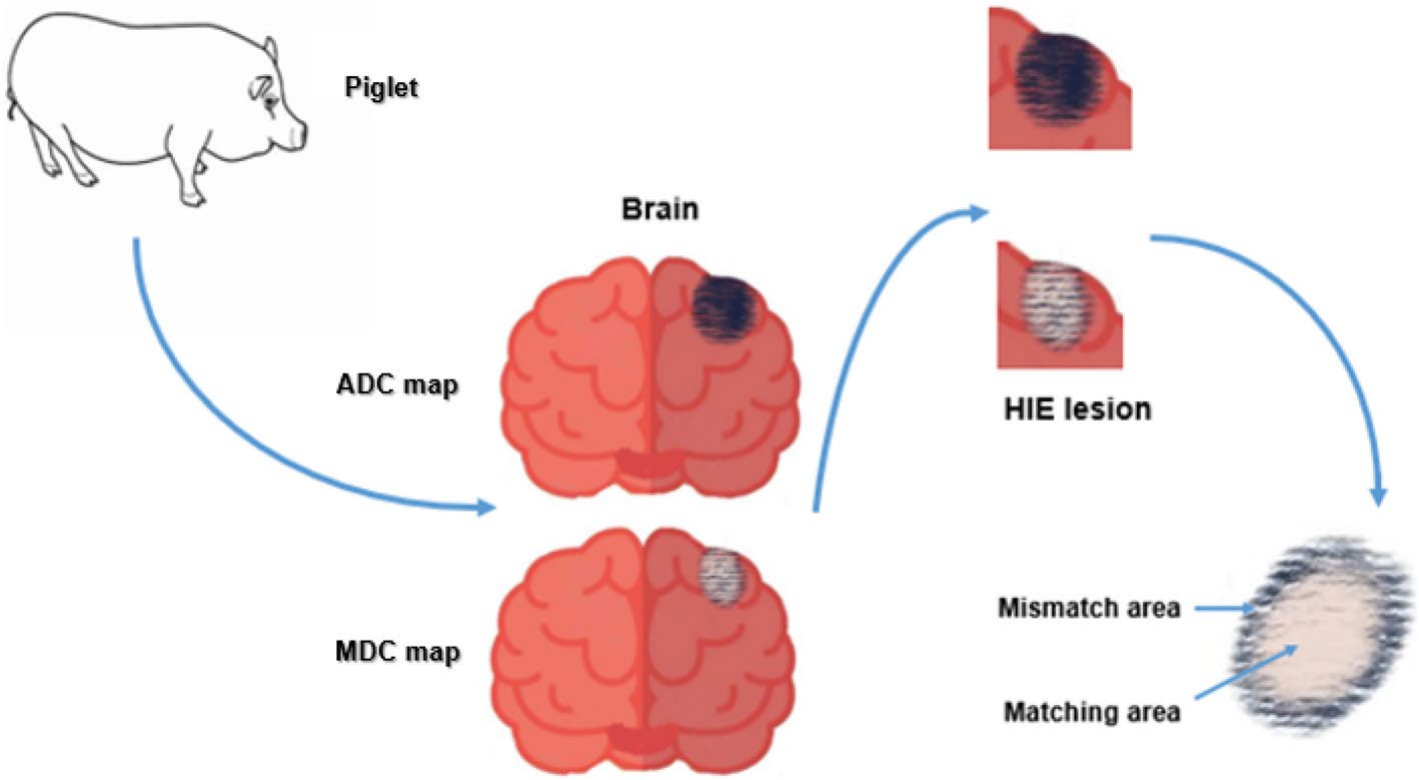
Schematic diagram of defining ADC/MDC matching area and ADC/MDC mismatching area

**Fig. 2:**
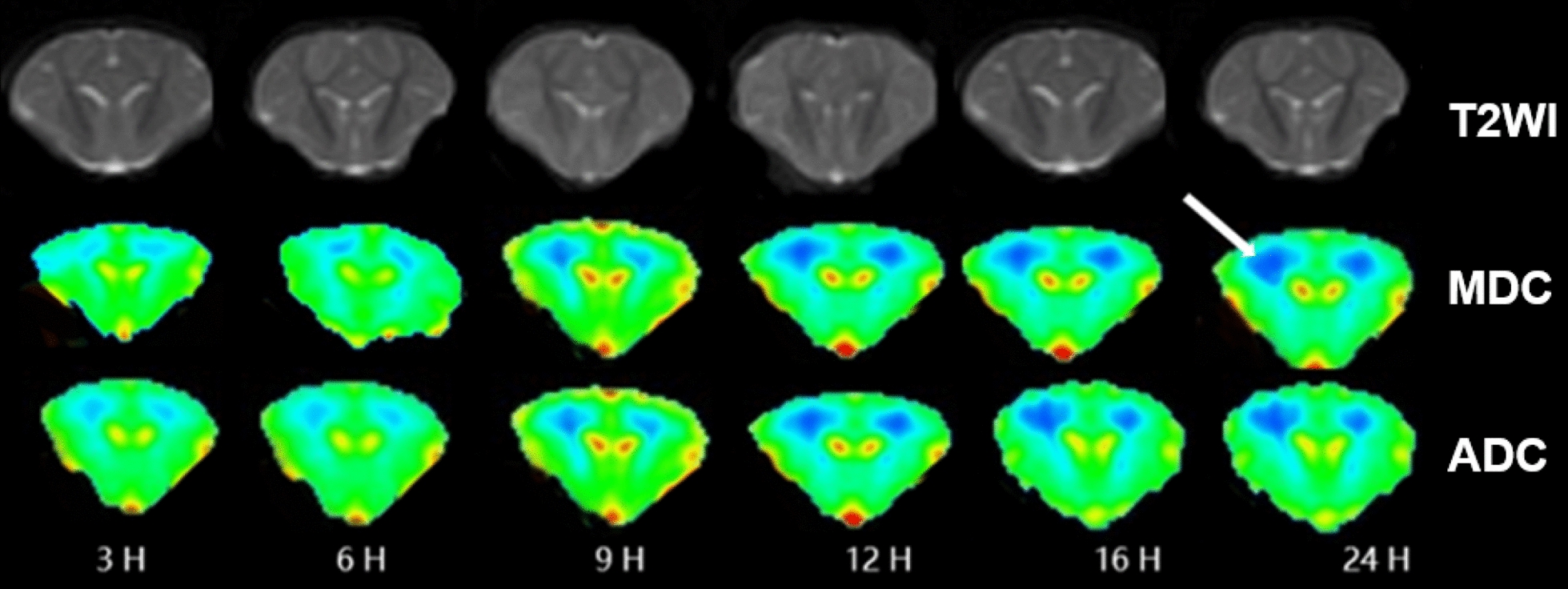
Changes in the MDC and ADC maps of the experimental group with time. The ADC, MDC and T2WI maps of the experimental group at different timepoints. Lesions were located in the subcortical region of the cerebral hemisphere and white matter around the ventricles (white arrow). ADC and MDC showed relatively uniform low signal (some dark blue area); T2WI signal does not change significantly

Figure 1 shows the flow chart of our study, and the ADC/MDC matching regions were defined as the area, where both the ADC and MDC showed abnormal signals, whereas the ADC/MDC mismatching regions were defined as the area, where the ADC showed abnormal signals, whereas the MDC showed normal signals. In this research, all pigs in the experimental groups developed lesions in the subcortical area and the white matter around the lateral ventricle. Figure 2 shows the MDC, ADC pseudocolor images and T2WI of each group at different timepoints. We found that in the experimental group, MDC and ADC map increased gradually with time (*P* < 0.05), and the signals of MDC and ADC maps were relatively homogeneous. However, there was no significant change on T2WI within 24 h (Fig. 2).

**Fig. 3:**
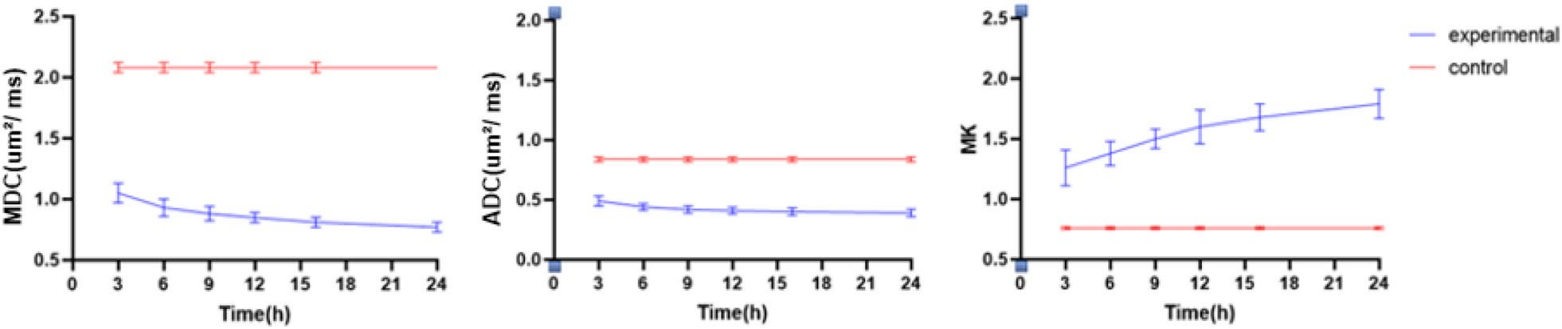
Time course of MDC, ADC and MK value between groups in the lesion areas

At each timepoint, the MK, AK and RK values (the parameters of the DKI) were significantly increased in the experimental group, whereas the MDC and ADC values were reduced (Table 1). The changes of MK, MDC and ADC values of the experimental group are shown in Fig. 3. The results showed that both MDC (the parameter of the DKI) and ADC (the parameter of the DWI) decreased within 24 h, while MK (the parameter of the DKI) values increased.

**Table 1:** DWI- and DKI-derived variables between groups at different timepoints

Time	Group	MK	MDC (μm^2^/ms)	ADC (μm^2^/ms)	AK	RK
3 h	Experimental	1.26 ± 0.15	1.05 ± 0.08	0.49 ± 0.04	1.19 ± 0.06	1.36 ± 0.09
	Control	0.76 ± 0.01	2.08 ± 0.04	0.84 ± 0.02	0.63 ± 0.02	0.86 ± 0.01
6 h	Experimental	1.38 ± 0.10	0.93 ± 0.07	0.44 ± 0.03	1.31 ± 0.07	1.45 ± 0.07
	Control	0.76 ± 0.01	2.08 ± 0.04	0.84 ± 0.02	0.63 ± 0.02	0.86 ± 0.01
9 h	Experimental	1.50 ± 0.08	0.88 ± 0.06	0.42 ± 0.03	1.42 ± 0.06	1.59 ± 0.06
	Control	0.76 ± 0.01	2.08 ± 0.04	0.84 ± 0.02	0.63 ± 0.02	0.86 ± 0.01
12 h	Experimental	1.60 ± 0.14	0.85 ± 0.04	0.41 ± 0.03	1.49 ± 0.10	1.67 ± 0.21
	Control	0.76 ± 0.01	2.08 ± 0.04	0.84 ± 0.02	0.63 ± 0.02	0.86 ± 0.01
16 h	Experimental	1.68 ± 0.11	0.81 ± 0.04	0.40 ± 0.03	1.53 ± 0.07	1.74 ± 0.08
	Control	0.76 ± 0.01	2.08 ± 0.04	0.84 ± 0.02	0.63 ± 0.02	0.86 ± 0.01
24 h	Experimental	1.79 ± 0.12	0.77 ± 0.04	0.39 ± 0.03	1.56 ± 0.07	1.86 ± 0.08
	Control	0.76 ± 0.01	2.08 ± 0.04	0.84 ± 0.02	0.63 ± 0.02	0.86 ± 0.01
*P*		< 0.001	< 0.001	< 0.001	< 0.001	< 0.001
P*		< 0.001	< 0.001	< 0.001	< 0.001	< 0.001
*P*^#^		< 0.001	< 0.001	< 0.001	< 0.001	< 0.001

*P*, Comparison between groups; P*, Among different timepoints; *P*^*#*^, Interaction of time × group

**Fig. 4:**
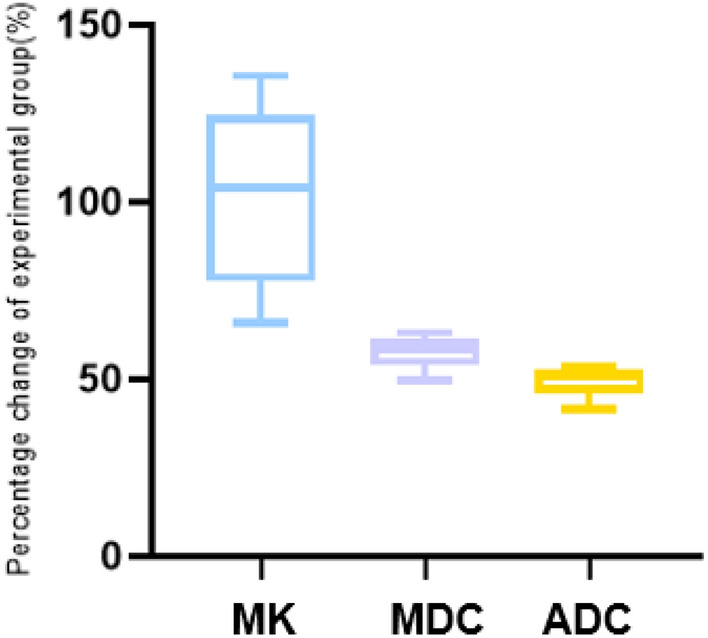
Percentage changes in DWI- and DKI-derived variables in the lesions

Interestingly, we found that the parameters of DKI (MDC and MK) changed rapidly from 3 to 12 h, while the parameter DWI (ADC) changed slowly during this period. However, both DKI parameters and DWI parameters changed slowly within 12–24 h (Fig. 3). The change in the percentage of MK and MDC was significantly higher than that of the ADC at different timepoints, and the rate of change of MK was higher than that of MDC in the experimental group (Fig. 4).

### Changes in lesion area

**Fig. 5:**
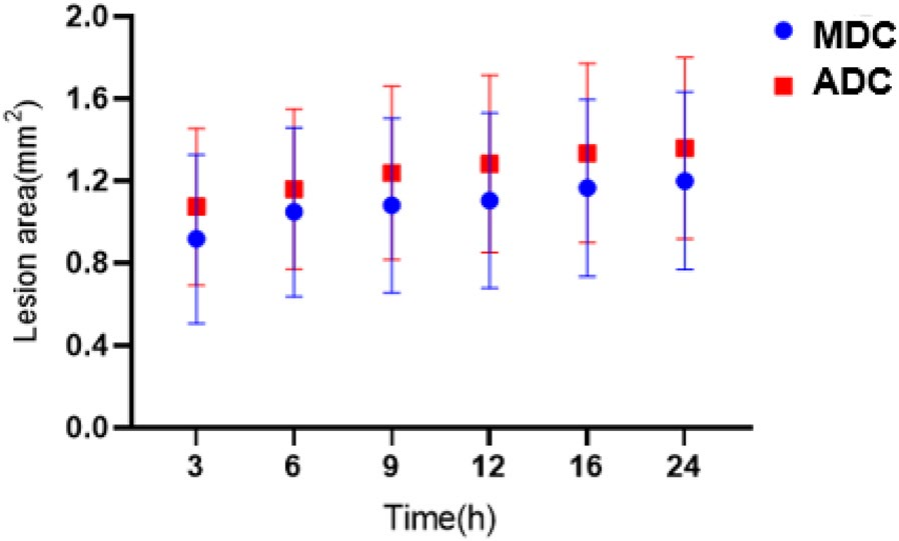
Area of ADC and MDC lesions in the experimental group over time

There were significant differences in the MDC and ADC lesions area between the experimental groups at each timepoint (*P* < 0.05) (Table 2). The MDC and ADC images showed obvious lesions at 3 h for the first time. At this time, the area of ADC lesions was larger than that of MDC. As the lesions developed, the area of ADC maps was always larger than that of the MDC maps within 24 h (Fig. 5).

**Table 2:** Comparison of ADC and MDC lesions area at different timepoints in the experimental group

Time	3 h	6 h	9 h	12 h	16 h	24 h	*P*
MDC (mm^2^)	0.917 ± 0.411	1.049 ± 0.411	1.081 ± 0.425	1.104 ± 0.425	1.165 ± 0.431	1.200 ± 0.433	< 0.05
ADC (mm^2^)	1.074 ± 0.380	1.160 ± 0.390	1.237 ± 0.423	1.282 ± 0.432	1.335 ± 0.438	1.359 ± 0.442	< 0.05
*P*	0.004	0.028	0.006	0.001	0.000	0.000	
*t*	*− *4.135	*− *2.775	*− *3.944	*− *5.164	*− *7.859	*− *6.103	

MDC and ADC represent lesions area (mm^2^) on the parameter maps; 3, 6, 9, 12, 16 and 24 h represent the time after hypoxia

### Pathological results

**Fig. 6:**
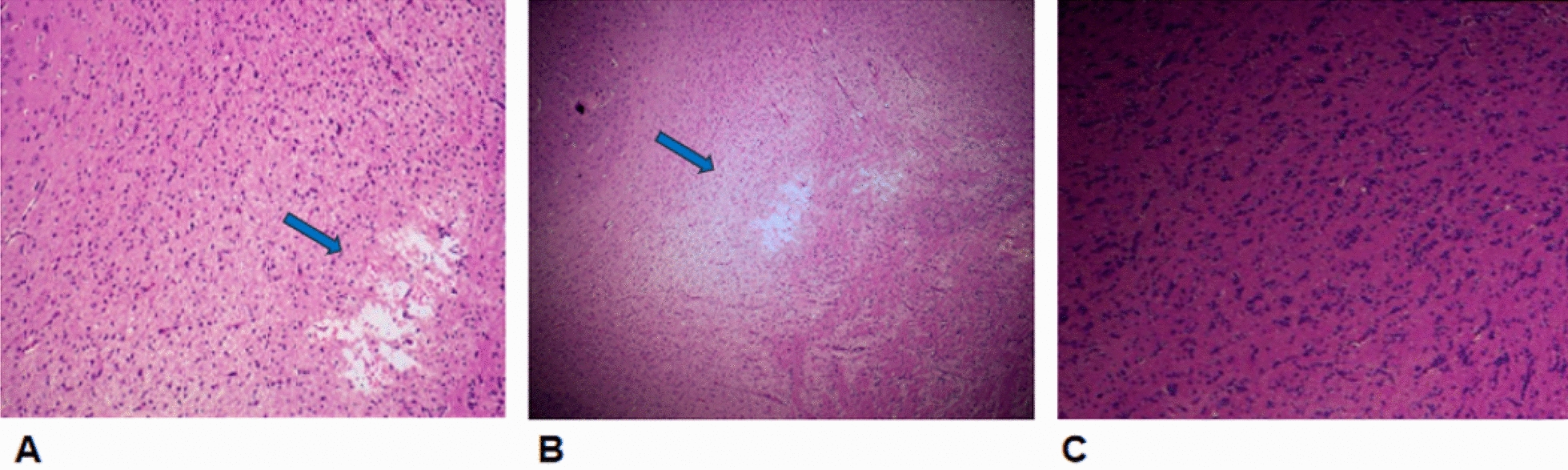
Optical microscope photograph between the groups at 24 h (HE 100×). **a, b** Pathological microscopic phenomenon of ADC/MDC matching area in the lateral ventricle area of the experimental group at 24 h. All of the glial cells were swollen, some of the nuclei were disintegrated and necrotic, and localized vasodilatation and congestion (blue arrow). **c** No abnormal pathological changes in the cells and intercellular spaces of the MDC/ADC mismatching areas

**Fig. 7:**
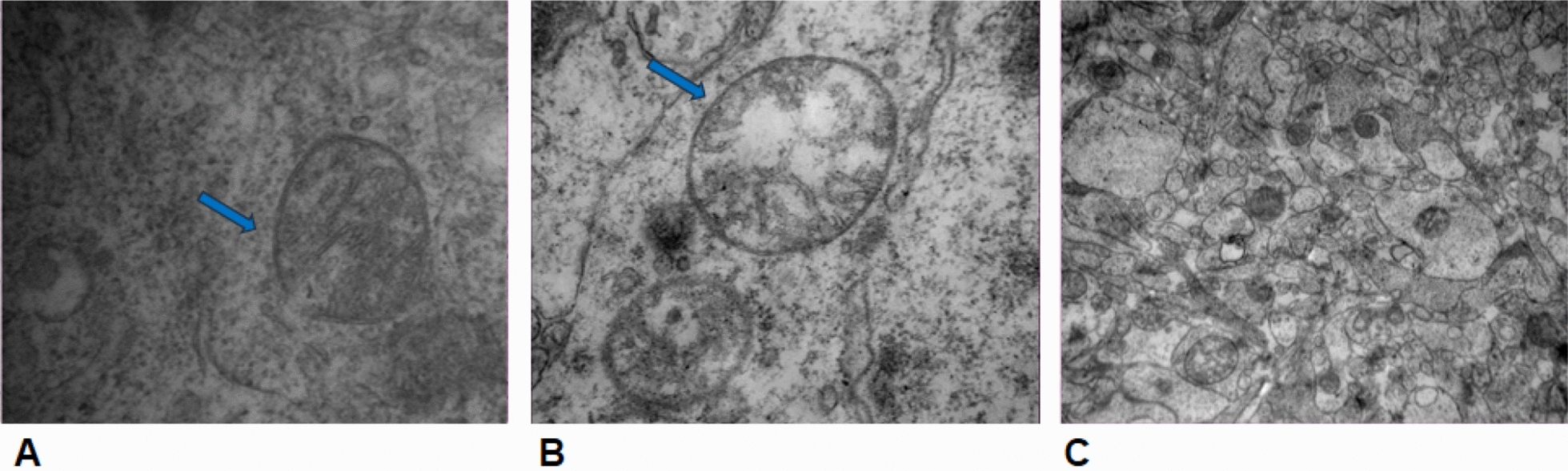
TEM photograph between the groups at 24 h (a: 100,000×, b: 80,000×, c: 25,000). **a, b** MDC/ADC matching area electron micrograph in the lateral ventricle area of the experimental group at 24 h, including mitochondria with different degrees of swelling, partial mitochondrial membrane collapse, rupture (blue arrow). **c** MDC/ADC mismatching area organelles were acceptable, and some were only slightly swollen

Under an optical microscope, compared with areas in the control group, some of the glial cell disintegration and necrosis areas were observed in the edge of lesions on the MDC/ADC matching areas of the experimental group. The surrounding glial cells were swollen, the intercellular space was widened and vasodilatation was observed locally (Fig. 6). In addition, there were no abnormal pathological changes in the cells and intercellular spaces of the MDC/ADC mismatching areas in the experimental group. Under an electron microscope, compared with areas in the control group, mitochondrial swelling, partial mitochondrial membrane collapse along with mitochondrial ridges fracture were observed in the edge of lesions on the MDC/ADC matching areas of the experimental group (Fig. 7). Interestingly, we found almost normal mitochondrial morphology in the ADC mismatching region under electron microscopy, suggesting that the ADC image region may be larger than the actual histological lesion region (Fig. 7).

## Discussion

Our study showed that with the development of HIE, the ADC and MDC values gradually decreased, whereas the MK, AK, and RK values gradually increased. In addition, the lesions continued to deteriorate, with a large change rate mainly in the first 12 h (MK, 1.105; MDC, 0.591; ADC, 0.512). It revealed that HIE progresses faster in the first 12 h, suggesting that intervention measures should be taken as soon as possible. Yang et al. also found that HIE lesions progressed most rapidly in the first 12 h [15]. It was previously found in the rat ischemic stroke model that lesions developed rapidly from 0.5 to 12 h, and the change rate of MK and MDC was always higher than that of ADC [16]. Consistent with previous findings, we also found that the rate of change of MK, MDC, and ADC was faster within 3–12 h, and the rate of change of MK and MDC was always higher than that of ADC. Therefore, the important parameters of DKI (MK and MDC) were more sensitive to the diagnosis of HIE injury than DWI (ADC), which was consistent with data from stroke studies [17], which could be defined as hypoxic–ischemic brain injury in adults.

Regarding to the lesion area of the HIE model, DWI and DKI depicted the lesion in the first scan of the experimental group, but conventional MR (T2WI) did not show it accurately (Fig. 2). With the development of HIE, the lesion area gradually increased, tissue damage gradually increased, and the lesion area gradually became stable after 16 h. We found that the lesion area presented by the ADC map at each timepoint was always larger than that of the MDC map (*P* < 0.05). Studies have found that in adults with ischemic stroke, the lesion area of the ADC map was 5.1% larger than that of the MDC map [17]. Combined with the pathological results, we found that the glial cells in the ADC/MDC matching area under the light microscope were swollen, some nuclei were decomposed and necrotic, and local vasodilation and hyperemia were observed. Under electron microscopy, the central cells and mitochondria in the ADC/MDC matching area of the lesion were swollen to varying degrees, the mitochondrial ridge was broken, the membrane collapsed, and the damage was severe. However, the cells and mitochondria in the ADC/MDC mismatching area were normal in shape and none of these morphological changes occurred. From the perspective of imaging principles, MDC, as a non-Gaussian corrected ADC, has more advantages. The DKI important parameter MDC based on the non-Gaussian distribution model of water molecule diffusion can more accurately reflect the true area of ​​HIE lesions. We have further confirmed at a pathological level that the ADC map exaggerated the range of HIE lesions.

The DWI parameter ADC is used to describe the diffusion movement of water molecule in different directions, and its calculation should use more than two different *b* values [18]. DKI introduces the fourth-order kurtosis defined in probability and statistics, which requires at least 15 directions and three *b* value calculations. The DKI parameter MDC is a comprehensive indicator to assess the diffusion status of a voxel, which reflects the overall situation of the molecular dispersion level and dispersion resistance. MDC is an ADC corrected by non-Gaussian distribution, which can more accurately reflect the ultrastructure of the cell, and is more sensitive to changes in the curvature of the cytoskeleton and cytoplasmic viscosity in the cell [19]. As a specific parameter of DKI, MK is more sensitive than ADC in reflecting the complexity of tissue microstructure. Our study has shown that DKI reflected more accurately pathological changes in damaged ischemic tissues, and its parameter MK and MDC signal change rates were also much higher than that of ADC, which can more sensitively diagnose the real extension of HIE lesion which DWI failed to find.

Our study has some limitations. For example, the sample size is small. Due to the limited tolerance of the animals, more frequent scans cannot be performed. In view of the fact that our research group previously studied the parameter MK (DKI), this study only compared the parameter MDC (DKI) with the parameter ADC (DWI). Based on previous studies on HIE, we studied only the important sites of HIE prevalence, including regions adjacent to the lateral ventricle and subcortical white matter. In the future, we will conduct studies on other sites, where lesions may occur.

## Conclusion

We used the newborn piglet as a model to compare the diagnostic ability of DKI and DWI for HIE. Our study revealed that DKI had the advantage of a more realistic image model, which could reflect the changes of brain microstructure more comprehensively and sensitively. Our research will have a broad application prospects in clinical work.

## Data Availability

The data sets used or analysed during the current study are available from the corresponding author on reasonable request.

## Abbreviations

DKI: Diffusion kurtosis imaging
HIE: Hypoxic–ischemic encephalopathy
DWI: Diffusion weighted imaging
MK: Mean kurtosis
AK: Axial kurtosis
RK: Radial kurtosis
MDC: Mean diffusion coefficient
ADC: Apparent diffusion coefficient
ROI: Region of interest
